# Artificial intelligence in paediatric neuroradiology: current landscape, challenges, and future directions

**DOI:** 10.1007/s00247-026-06547-9

**Published:** 2026-02-23

**Authors:** Brendan S. Kelly, Simon M. Clifford, Kshitij Mankad, Gabrielle C. Colleran

**Affiliations:** 1 Department of Radiology, Children’s Health Ireland at Crumlin, Cooley Rd, Dublin, D12 N512 Ireland; 2 School of Medicine, University College Dublin, Dublin, Ireland; 3 Clinical Neuroradiology, Great Ormond Street Hospital, London, United Kingdom; 4 Medicine, University College London, London, United Kingdom; 5 Department of Radiology, National Maternity Hospital, Dublin, Ireland; 6 Paediatrics, Trinity College Dublin, Dublin, Ireland

**Keywords:** Artificial intelligence, Fetal imaging, Neuroradiology, Paediatric radiology

## Abstract

This narrative review maps the current landscape of artificial intelligence (AI) in paediatric and fetal neuroradiology, critically evaluating current practice, barriers to clinical adoption, and future potential. We searched for peer-reviewed studies from the last decade, focusing on image segmentation, lesion detection, classification, prognostication, and clinical decision support in paediatric brain imaging. Particular consideration was given to unique paediatric factors such as brain development and data scarcity. AI techniques, notably deep learning, have demonstrated success in automated brain tumour segmentation, detection of epileptogenic lesions, and radiomics-based classifiers predicting tumour histology and molecular subtypes. Despite these advancements, clinical adoption remains limited. Key barriers identified include high implementation costs, limited large-scale diverse paediatric datasets, and concerns regarding safety, bias, and regulatory approval. Addressing these issues through data-sharing initiatives, federated learning, paediatric-specific validation, and revised ethical and regulatory frameworks is crucial. Ongoing multi-institutional collaborations can facilitate AI’s integration into paediatric neuroradiology, complementing radiologists and improving paediatric care.

**Graphical Abstract:**

**Figure:**
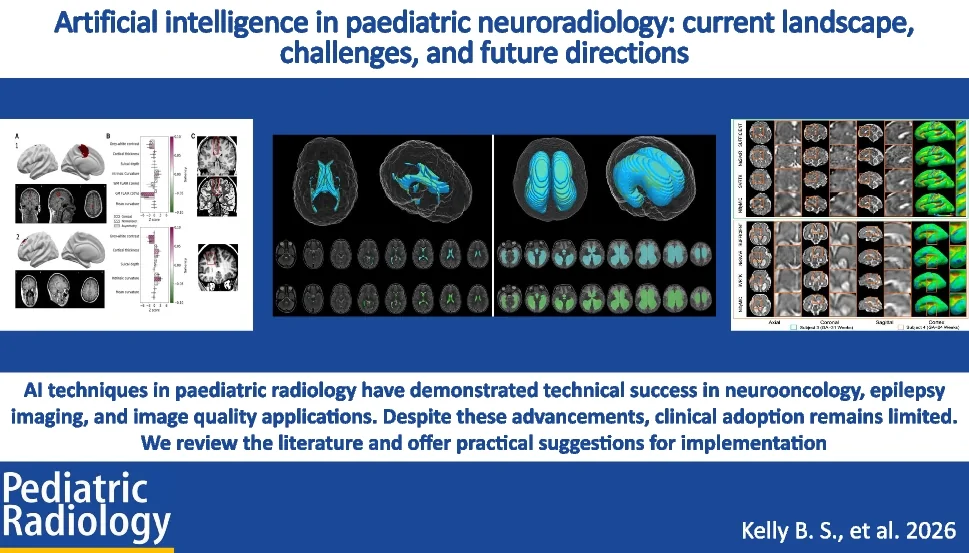

## Introduction and background

Paediatric and fetal neuroradiology encompasses imaging of the developing brain and nervous system, presenting distinct challenges from adult neuroradiology due to rapid brain maturation, evolving myelination, and age-specific conditions. Differentiating normal developmental variations from pathology, such as delayed myelination vs. leukodystrophies, on magnetic resonance imaging (MRI), can be particularly challenging. Young children often require sedation for diagnostic quality MRI, while computed tomography (CT) necessitates strict adherence to As Low As Reasonably Achievable (ALARA) principles to minimise radiation exposure. Additionally, congenital anomalies (including cortical malformations) and rare genetic syndromes (including leukodystrophies) require specialised expertise.

These unique aspects underpin both the rationale and challenges for artificial intelligence (AI) in paediatric neuroradiology. Modern multi-parametric imaging techniques (e.g. diffusion and perfusion MRI) provide extensive data but risk overwhelming radiologists [1–4]. AI algorithms, including machine learning (ML) and deep learning, excel at detecting complex patterns in high dimensional datasets, for example, models trained on multi-parametric paediatric brain MRI can integrate features across several sequences to segment brain tumours or highlight subtle focal cortical dysplasia that may be difficult to appreciate on routine visual review. AI could synthesise large imaging and clinical datasets, supporting diagnosis and prognostication where traditional interpretation is challenged [1, 5, 6]. Importantly, AI may address longstanding paediatric issues by facilitating shorter MRI protocols or correcting motion artefacts to avoid sedation and optimising CT radiation doses intelligently [7–11]. AI can further support radiologists by identifying subtle, age dependent imaging features in childhood tumours or epilepsy, correlating imaging with clinical and genomic data for personalised medicine [1, 12]. Due to the rarity of many paediatric neurological disorders and limited datasets, a comprehensive evaluation of AI research is essential [13]. Herein, we present a narrative summary of a 10-year scoping review of the AI in paediatric neuroradiology literature. This review highlights AI’s transformative potential in paediatric neuroradiology, leveraging its strengths in data integration, pattern recognition, and automation to enhance diagnostic accuracy and patient safety while also considering challenges for discovery research and barriers to implementation adoption.

## Information sources and search strategy

We conducted a structured narrative review to map the landscape of AI applications in paediatric and fetal neuroradiology. The search covered the period 1 January 2015 to 31 December 2025, with a final search performed on 27 January 2026 to ensure completeness.

Searches were performed across PubMed, Google Scholar, medRxiv, arXiv, and major imaging journals and society platforms. Preprints were considered eligible when explicitly labelled. The search combined seeded queries, iterative query expansion, and snowballing (both backward and forward citation chasing).

An example Google Scholar query was:

- (“paediatric” OR “pediatric”) AND (neuroradiology OR “brain MRI” OR “head CT”) AND
- (“artificial intelligence” OR “machine learning” OR “deep learning”)

For PubMed, we focused on titles and abstracts, incorporating MeSH terms where available:

- ((“paediatric”[tiab] OR “pediatric”[tiab]) AND (neuroradiology[tiab] OR brain[tiab]) AND
- (“artificial intelligence”[tiab] OR “deep learning”[tiab] OR “machine learning”[tiab]))

Citation snowballing was repeated for all included studies to identify additional relevant work. Studies were included if they met the following criteria:

- Population: paediatric cohorts (0–17 years) or mixed populations with extractable paediatric data
- Domain: neuroradiology imaging tasks
- Methods: applications of AI, including deep learning and machine learning approaches
- Study types: original studies, reviews, technical guidance, or consensus documents relevant to clinical practice
- We did not explicitly exclude studies reporting non-imaging AI applications or opinion pieces but prioritised original research in our synthesis.

Title and abstract screening was conducted by a single reviewer, followed by full-text assessment. Findings were synthesised narratively, structured by modality and task. We placed particular emphasis on dataset provenance, external validation, clinical integration, and readiness for implementation. While preprints were included to capture emerging developments, conclusions relied preferentially on peer-reviewed evidence.

We aligned the review process with best-practice recommendations for narrative reviews, following the scale for the assessment of narrative review articles (SANRA) framework to ensure clarity, transparency, and scientific rigour. Specifically, we addressed SANRA’s [14] six items: importance, aims, literature search, referencing, scientific reasoning, and presentation of endpoint data prospectively from the outset of our search by design.

## Current state of artificial intelligence in paediatric neuroradiology

AI research in paediatric neuroradiology has expanded, although it remains less developed than adult imaging. Studies typically focus on tasks such as image segmentation, lesion detection, classification, and outcome prediction, centred around specific paediatric neurological conditions. Table 1 summarises representative AI applications and outcomes in paediatric neuroradiology.

**Table 1:** Representative artificial intelligence applications in paediatric neuroradiology

Study (year)	Focus (disease/task)	AI method	Key finding/outcome	Limitations
Jeong et al. (2022) [15]	Paediatric epilepsy (MRI negative cases)	Multi-modal deep CNN	Detected previously occult epileptogenic foci in ~15% of drug-resistant epilepsy patients with initially normal MRI	Retrospective and observational study. Small sample size (*n*=41). Selection bias (specialised population)
Quon et al. (2020) [16]	Hydrocephalus (ventricle segmentation)	3-Dimensional (D) CNN segmentation	Automated segmentation of cerebral ventricles for volume calculation, aiding paediatric hydrocephalus evaluation	Retrospective and observational study. Volume only – does not classify hydrocephalus. Selection bias (normal only)
Yeom et al. (2022) [17]	Medulloblastoma (tumour subtyping)	MRI radiomics+machine learning	Noninvasively classified 4 molecular subgroups of medulloblastoma from MRI with high accuracy (radiogenomic profiling)	Retrospective. Heterogeneous MRI scans were normalised. Only presurgical imaging without diffusion, fingerprinting, or perfusion. No spatial context
Vecchiato et al. (2021) [18]	Non-sedated paediatric MRI (motion artefact)	Retrospective motion correction	AI-driven k-space reordering (DISORDER) significantly improved MRI image quality and diagnostic readability in 75% of scans without sedation	*n*=32. Longer scan times. Their methods increase motion artefact and before subsequent correction. Impact of reconstruction delay (up to 1 h) and a quantification of maximum tolerable degradation not assessed
Chang et al. (2021) [19]	Paediatric brain tumours (prognostication)	Radiomic feature analysis	Identified MRI texture features correlating with tumour molecular subtypes; certain features predicted poorer survival (*P*<0.05)	Retrospective. *n*=27. Limited accuracy. Limited images only used

*3-D* three-dimensional, *AI* artificial intelligence, *CNN* convolutional neural network, *MRI* magnetic resonance imaging, *n* sample size number

**Fig. 1:**
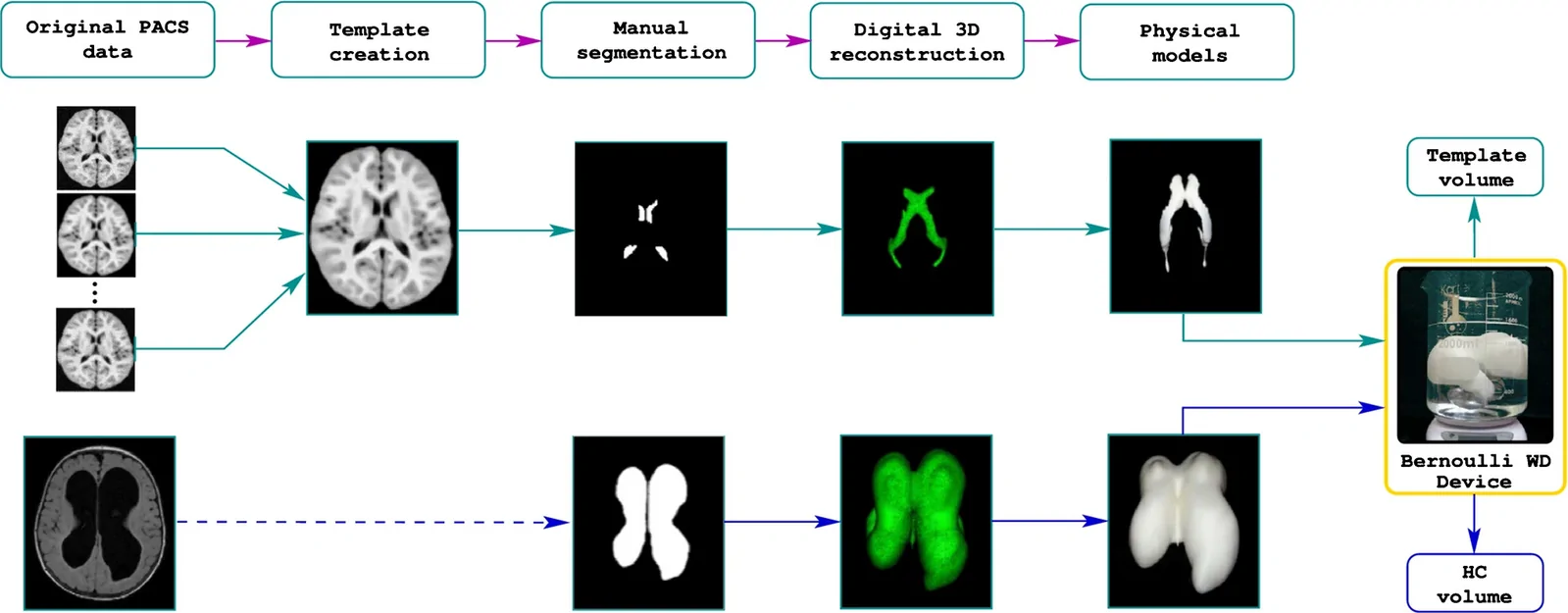
A segmentation and 3-dimensional printing pipeline for ventriculomegaly via deep learning. From medical images, the lateral ventricles’ masks are obtained. Processed masks are transformed into physical models. Reproduced with permission under the open-access terms of the Nature Portfolio journals [20]

Early AI successes in paediatric neuroradiology have primarily involved segmentation and quantitative analysis [5, 6]. Deep learning models have been used effectively to segment paediatric brain structures and lesions on MRI. For example, automatic ventricular segmentation in hydrocephalus enables precise volume measurement [9, 16]. Quon et al. developed an AI tool segmenting cerebral ventricles on MRI/CT to track hydrocephalus progression objectively [16]. These techniques have also been combined with 3-dimensional printing (Fig. 1) [20]. Tumour segmentation, particularly using U-Net-based models, is another significant area, generating reproducible tumour volumes in paediatric gliomas and medulloblastomas [17]. The recent BraTS-PEDs challenge highlights international efforts to benchmark paediatric tumour segmentation using multi-centre datasets [21].

**Fig. 2:**
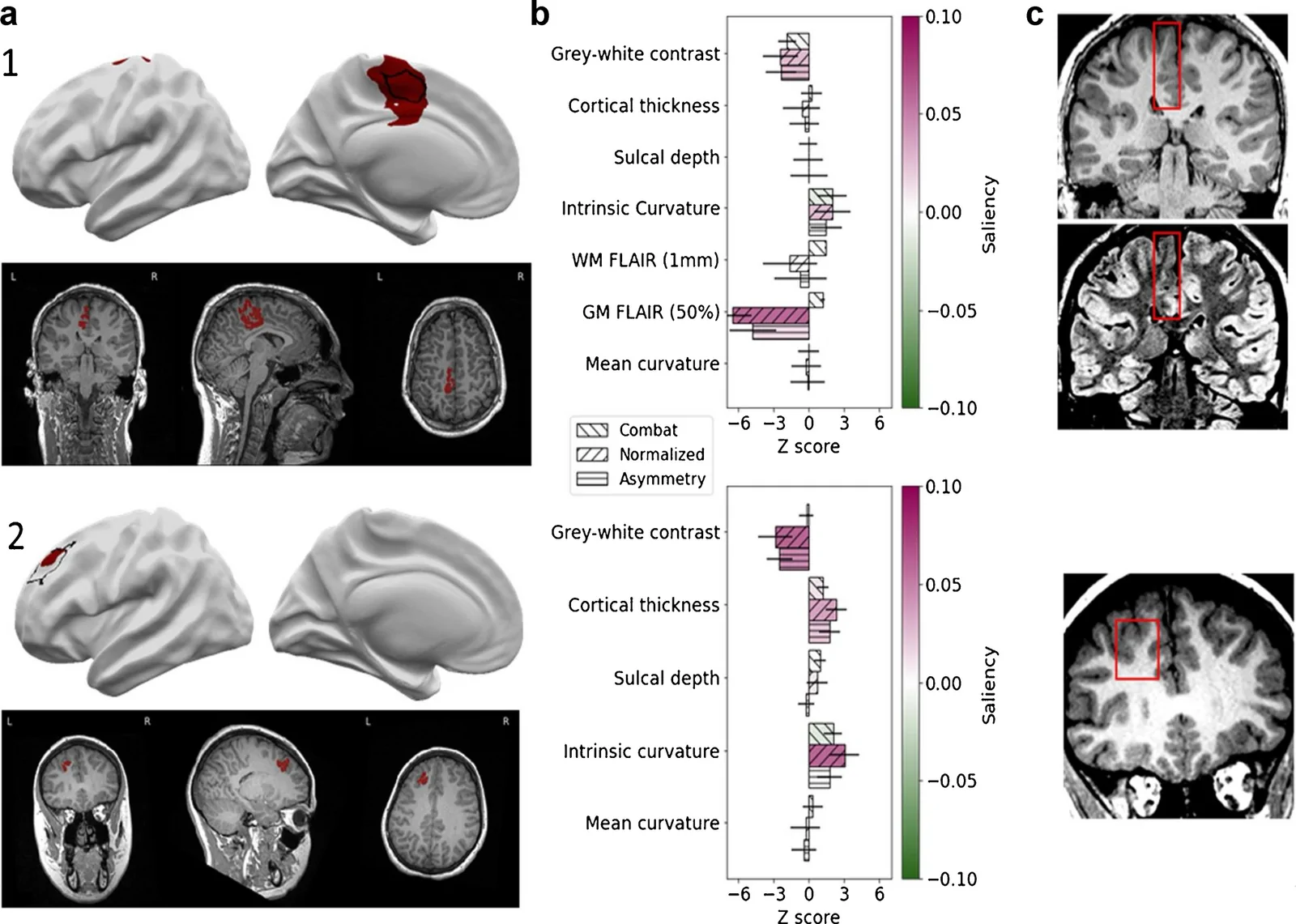
Example classifier predictions with saliency scores for “Patient 1” (an example with fluid attenuation inversion recovery (FLAIR) data) and “Patient 2” (without FLAIR data). **a** Classifier predictions (*dark red*) and manual lesion mask (*black line*) visualised on brain surfaces (only lesional hemisphere is shown). Classifier predictions (*dark red*) visualised on T1 volume. **b** *Z*-scored mean feature values within predicted lesions coloured with integrated gradients saliency scores. Positive saliency scores indicate feature values driving the classifier’s “lesion” prediction. Negative scores indicate feature values that are inconsistent with the prediction. **c** Lesional cortex highlighted on the patients’ scans exhibits salient features automatically identified by the classifier. Reproduced from [22] under the terms of the *Creative Commons CC BY* license

AI-driven lesion detection and classification methods are also emerging, especially in epilepsy and tumour characterisation. In paediatric epilepsy, subtle cortical malformations, often undetectable on standard MRI, are a significant challenge (Fig. 2) [22]. Jeong et al. reported a deep learning model using multi-modal MRI that localised epileptogenic zones in drug-resistant epilepsy, identifying lesions missed on initial MRIs in 15% of cases [15]. The Multi-Centre Epilepsy Lesion Detection (MELD) group and their recent extension using graph neural networks also continue to make significant contributions to the field [22–24]. Additionally, prototype tools have been developed for paediatric head CT, targeting automated detection of intracranial haemorrhage or skull fractures, though robust paediatric validation remains limited compared to adult studies [25, 26].

**Fig. 3:**
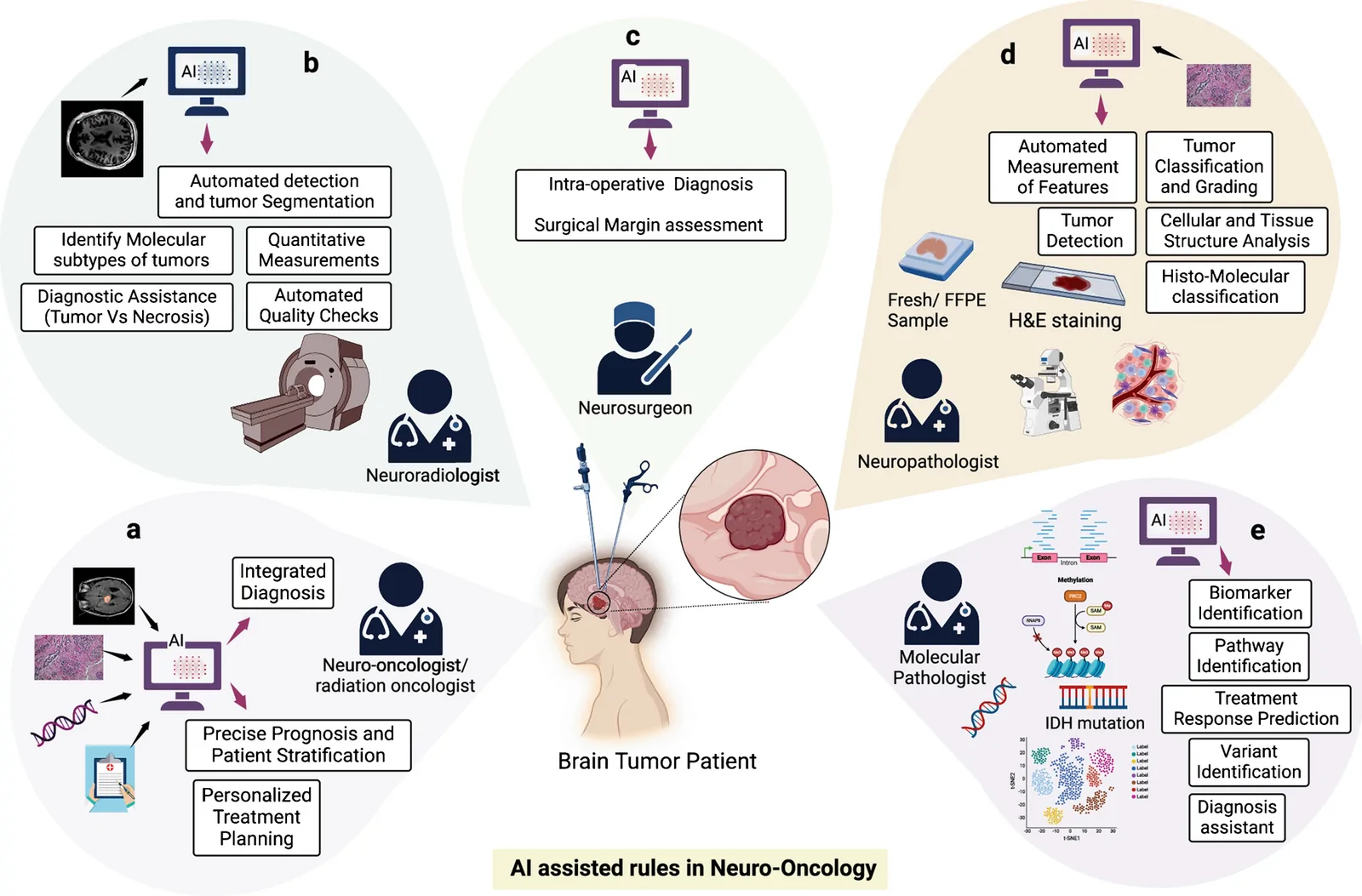
**a** Artificial intelligence (AI) augments the capabilities of neuro-oncologists/radiation-oncologists by enabling integrated diagnosis, offering deeper insights into the disease, facilitating precise prognosis by predicting outcomes, and assisting in patient stratification to tailor treatment plans to individual needs. **b** AI supports neuroradiologists by leveraging images for automated detection and tumour segmentation, identifying molecular subtypes of tumours, providing quantitative measurements, and delivering diagnostic assistance to distinguish tumours from necrotic regions, all while ensuring automated quality checks. **c** AI aids neurosurgeons during surgery, contributing to surgical margin assessment and offering real-time diagnosis information and guidance, enhancing surgical precision and patient outcomes. **d** AI assists neuropathologists in the analysis of fresh/frozen samples, providing automated measurement of features, aiding in tumour classification and grading, improving tumour detection, and delivering comprehensive analysis of cellular and tissue structures through histo-molecular classification. **e** Handling mutation data, single-cell information, methylation patterns, RNA sequencing, and more, AI empowers molecular pathologists by supporting biomarker identification, pathway identification, treatment response prediction, variant identification, and serving as a diagnosis assistant, streamlining the complex molecular analysis process (created with BioRender.com). Reproduced from [29] with permission under the open-access terms of the Nature Portfolio journals

Radiomics (quantitative imaging feature extraction) methods have shown promise for classification and outcome prediction, especially when combined with machine learning. This has been used notably in paediatric neurooncology [1, 8, 12, 17]. Radiomic features can be correlated with clinical or genomic data, enabling non-invasive tumour characterisation. Yeom et al. demonstrated that MRI-based ML models accurately differentiate molecular subgroups of medulloblastoma (Wingless/WNT, Sonic Hedgehog/SHH, Group 3, Group 4), with area under the curve values between 0.80–0.90 [17]. Chang et al. similarly reported MRI texture features differentiating medulloblastoma subgroups and predicting survival outcome [19]. Additionally, ML has been utilised to estimate gestational age or brain maturity from neonatal MRI and to predict neurodevelopmental outcomes [27, 28]. These studies underscore AI’s potential to reveal subtle prognostic imaging biomarkers beyond visual assessment, as illustrated in Fig. 3.

Overall, existing literature demonstrates proof-of-concept successes in paediatric neuroradiology AI. However, studies typically involve small retrospective cohorts (often tens to low hundreds) [5, 6] due to the rarity of paediatric conditions and barriers to multi-centre data sharing. Compared to adult neuroradiology, paediatric AI applications remain primarily in the research domain, with limited clinical deployment and widespread calls for further validation [1, 11, 13].

## Current state of artificial intelligence in fetal neuroimaging

**Fig. 4:**
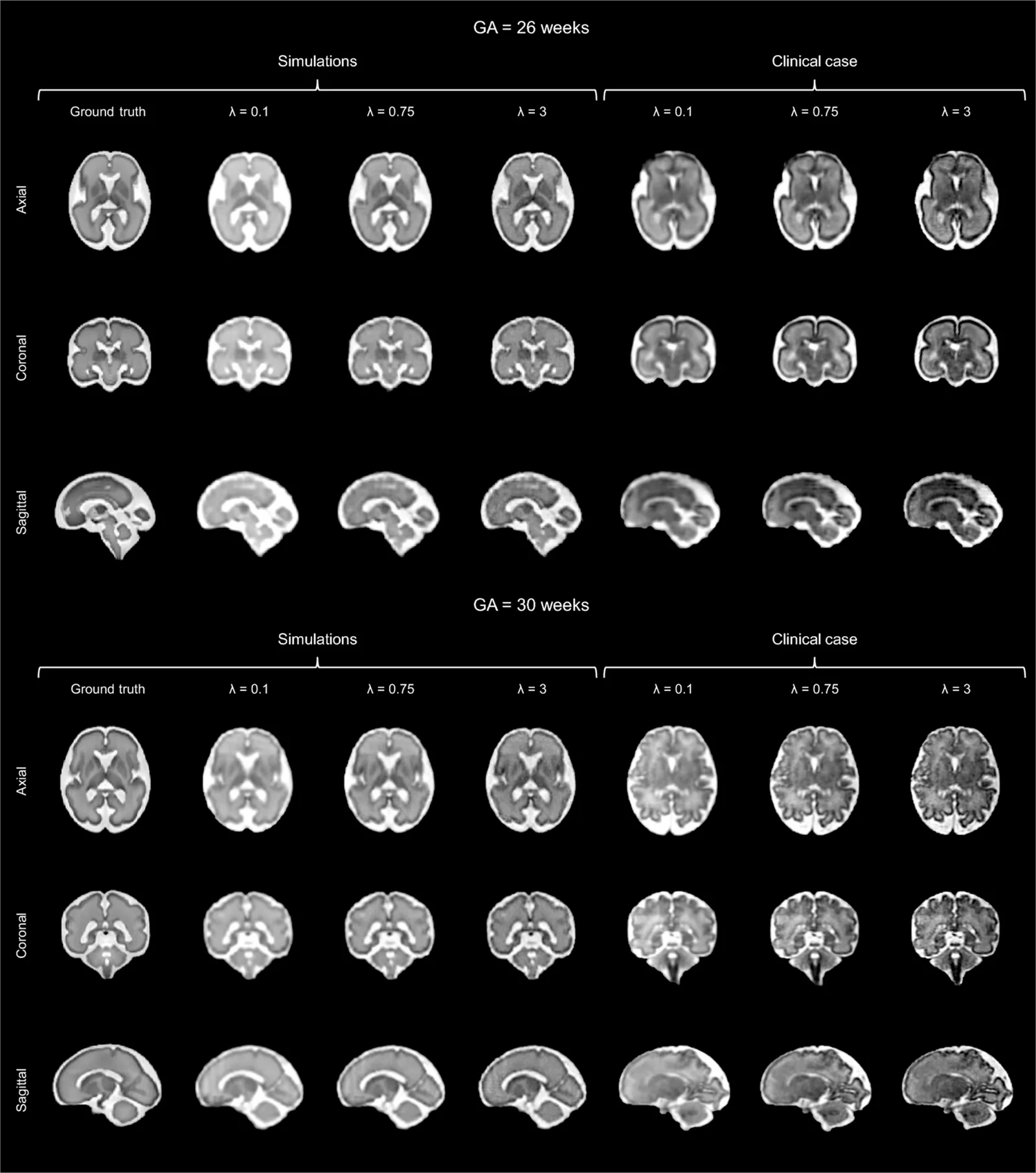
Appreciation of the quality of reconstruction depending on the weight (*W*) that controls the strength of the regularisation. The framework for optimising the reconstruction quality through parameter fine-tuning in the presence of motion is illustrated at two gestational ages: 26 weeks and 30 weeks. Two representative clinical cases are provided for comparison. The results for three values of *W* are presented. For *W*=0.1, the reconstruction looks blurry with poor tissue contrast. Using W=3 improves the contrast but the images look noisy. For *W*=0.75, the reconstruction is sharp, with a contrast between different brain tissues similar to that observed in the isotropic ground truth. Clinical cases from which the simulated images are derived highlight the accuracy of a reconstruction for this intermediate value of *W*, especially with regard to the definition of the corpus callosum and the delineation of the cortex. These reconstructions have been shown to aid artificial intelligence segmentation, a technologic synergy. Reproduced from Wu et al. with permission under the open-access terms of the Nature Portfolio journals [30]

Fetal brain imaging provides crucial prenatal insights but is technically demanding due to fetal motion, evolving neuroanatomy, and variability in image quality. AI methods, particularly deep learning, are addressing these longstanding obstacles with the potential to transform prenatal diagnosis and management. Clinically relevant AI applications include detailed anatomical segmentation, robust motion correction, early anomaly detection, accurate gestational age estimation, and potential prediction of neurodevelopmental outcomes. Wu et al. [30] provide an example of an AI used for multi-planar reconstruction. Potential application of reconstruction techniques and their synergy with AI is shown in Fig. 4 [31]. These developments offer promising avenues for improved diagnostic precision and informed parental counselling in clinical fetal neuroradiology practice.

Automated segmentation via convolutional neural networks (CNNs) has substantially enhanced fetal brain MRI quantification, providing volumetric metrics and morphometric analysis even in complex clinical scenarios. Models such as multi-scale CNNs and 3-dimensional U-Net architectures now achieve consistent segmentation accuracy (Dice≥0.90) in both normative and pathological conditions, including fetuses with congenital brain anomalies or ventriculomegaly [32]. The clinical impact of this is notable, allowing neuroradiologists to objectively monitor ventricular dilation or cortical abnormalities longitudinally, facilitating early intervention planning. However, substantial technical challenges persist, notably dataset scarcity, cross-institutional heterogeneity, and limited external validation, underscoring the importance of robust multi-centre collaborations for future clinical translation [33]. Complementary AI-driven motion correction techniques, using unsupervised learning frameworks and generative adversarial networks (GANs), now offer reliable retrospective mitigation of motion-induced image degradation. Such advancements can reduce repeat examinations and sedation requirements, potentially improving patient safety and clinical workflow [30, 34, 35]. Nevertheless, these methods currently remain primarily within research contexts and demand rigorous clinical validation and integration into diagnostic imaging protocols to establish real-world utility [30, 35].

In fetal neurosonography, CNNs enhance screening by automatically verifying critical standard planes (transventricular, transcerebellar), promoting more consistent anatomical assessment. Furthermore, AI classifiers effectively distinguish normal from abnormal brain ultrasounds (accuracy ~96%), generating lesion-localising heatmaps that guide clinicians towards regions warranting detailed examination [36, 37]. The clinical utility of such approaches is evident, potentially reducing inter-operator variability, minimising missed diagnoses of subtle abnormalities, and improving referral decisions. Similarly, AI-driven gestational age prediction from fetal MRI images, leveraging sophisticated attention-guided deep-learning networks, demonstrates accuracy within approximately 1 gestational week, outperforming traditional biometric methods particularly during advanced gestation [38, 39]. In clinical practice, this precise developmental staging could identify subtle deviations from expected brain maturation, signalling neurodevelopmental vulnerability, particularly in fetuses exhibiting ventriculomegaly or other structural anomalies [40]. Preliminary investigations into using prenatal cortical expansion metrics from MRI for postnatal neurodevelopmental prognosis indicate a promising clinical horizon, although large-scale longitudinal validation is still required, especially in the context of the required follow-up duration (as neurodevelopmental outcomes often evolve beyond early childhood) [41, 42]. Ultimately, however, these remain primarily research applications and transitioning such AI tools from research to routine clinical care demands systematic evaluation through prospective multi-centre studies, rigorous external validation, and explainable AI methodologies that ensure clinical acceptability, transparency, and trustworthiness.

## Current clinical application

Many emerging AI applications in paediatric neuroradiology are already being prototyped or tested in clinical research settings. Table 2 in this review lists available AI devices relevant to paediatric neuroimaging, highlighting the limited number explicitly designed or validated for this population. Note that these devices are advertised as applicable to children or across all ages, even though some of the use cases do not appear to be directly relevant to children, e.g. dementia and coronary artery disease. Available data was taken in good faith, as it was presented on radiology.healthairegister.com. A recent federated learning model, FL-PedBrain, is an emerging example. It successfully classified and segmented paediatric brain tumours across 19 institutions, demonstrating performance comparable to traditional centralised training [44]. This real-world study illustrates how federated learning circumvents data-sharing barriers by “bringing AI to the data”. Similarly, an AI model analysing neonatal MRI accurately predicted developmental maturity and identified infants at risk for developmental delays, facilitating earlier interventions [28].

**Table 2:** Radiology AI Devices whose Intended use incudes both neuroradiology and either all ages or children. Reproduced from Kelly et al 2025 [43]

Product name	Company	Modality	Disease targeted	Key-features	Suggested use (before/during/after interpretation)
AI-Rad Companion Brain MR	Siemens Healthineers	MR	Neurodegenerative disease, Multiple Sclerosis	Brain volume quantification, segmentation, normative comparison, white matter hyperintensities, report generation, longitudinal comparison	During: perception aid (prompting all abnormalities/results/heatmaps)
IB Delta Suite	Imaging Biometrics	MR	Neuro lesions	Image standardisation, Delta T1 maps	
JAD-02K	JLK Inc.	MR	Dementia	Cortical area segmentation, cortical thickness quantification, brain aging status, normal comparison	During: perception aid (prompting all abnormalities/results/heatmaps), report suggestion
JBA-01K	JLK Inc.	MR	Cerebral aneurysm	Cerebral aneurysm detection	During: perception aid (prompting all abnormalities/results/heatmaps), report suggestion
NeuroQuant	Cortechs.ai	MR	Neurodegenerative brain disorders	Brain region quantification (75 structures), brain change data, normative data comparisons, dynamic atlas, custom report options	Before: flagging acute findingsDuring: perception aid (prompting all abnormalities/results/heatmaps), interactive decision support (shows abnormalities/results only on demand)
NeuroreaderÆ	Brainreader	MR	Neurodegenerative disorders	Brain region quantification, comparison to a normative database	Diagnostic aid
Pixyl.Neuro.FL	Pixyl	MR	Leukoencephalopathy, vascular dementia, small-vessel disease	White matter hyperintensities detection, white matter hyperintensities volume quantification, longitudinal analysis	During: perception aid (prompting all abnormalities/results/heatmaps)
Pixyl.Neuro.MS	Pixyl	MR	Multiple Sclerosis	Lesion detection, lesion quantification, lesion classification, lesional volume quantification, disease activity, longitudinal analysis, image annotation, McDonald MRI criteria	During: perception aid (prompting all abnormalities/results/heatmaps), report suggestion After: diagnosis verification
CoLumbo	Smart Soft Healthcare	MR	Disk herniation (Protrusion/Extrusion modifiers), Central Spinal Stenosis (CSS), Shizas and Lee morphological grading, decreased vertebral body heights, decreased disk height, hypo/hyperlordosis, nerve root impingement, listhesis	Lumbar spine annotation, smart navigation, editable report, editable separate pathology report, measurement calculation based on segmentation	During: perception aid (prompting all abnormalities/results/heatmaps), interactive decision support (shows abnormalities/results only on demand), report suggestion
AIRAscore structure	AIRAmed	MR	Neurodegenerative diseases, e.g. dementia, Alzheimer’s disease, multiple sclerosis, Parkinson’s Disease	Brain tissue and anatomy segmentation, volume quantification, normative comparison, report generation, WMH detection and quantification	During: perception aid (prompting all abnormalities/results/heatmaps), interactive decision support (shows abnormalities/results only on demand)
Annalise Enterprise CTB	annalise.ai	CT	130 findings present in the emergent, urgent, and non-urgent care settings including: Intracranial haemorrhage, haematomas (acute subdural/extradural), fractures, pneumocephalus, infarct, mass effect & midline shift, obstructive hydrocephalus, Intraaxial mass, transependymal oedema, colloid cyst, vasogenic oedema, and many others.	Detection of up to 130 findings (acute and chronic) on brain CT, worklist triage, notification, visualisation, confidence bar, normal/abnormal differentiation	Before: adapting worklist order, flagging acute findings During: perception aid (prompting all abnormalities/results/heatmaps), interactive decision support (shows abnormalities/results only on demand)
Brain aneurysm (BA)	Aidoc	CT	Brain aneurysm	Triage tool, workflow improvement	Triage/prioritisation of medical images
CINA-ASPECTS	Avicenna.AI	CT	Stroke	Automated ASPECT score, visual localisation information	During: perception aid (prompting all abnormalities/results/heatmaps)
e-ASPECTS	Brainomix	CT	Stroke	ASPECTS score, ischemic volume quantification, HDVS detection, hyperdensity detection and quantification, report generation	
icobrain ms	icometrix	MR	Multiple sclerosis	White matter abnormality quantification, brain volume change, report generation	During: perception aid (prompting all abnormalities/results/heatmaps), report suggestion
icobrain tbi	icometrix	CT, MR	Traumatic brain injury	Volume quantification, brain volume changes, report generation, cisternal compression, midline shift, mass lesions, traumatic axonal injuries	During: perception aid (prompting all abnormalities/results/heatmaps), report suggestion
Intracranial Hemorrhage (ICH)	Aidoc	CT	Intracranial Hemorrhage	Triage tool, workflow improvement	Triage/prioritisation of medical images.
Ischemic Stroke	Aidoc	CT	Large vessel occlusion (M1, ICA), medium vessel occlusions (M2), posterior occlusions	Triage tool, workflow improvement	Triage/prioritisation of medical images.
Neurocloud SISCOM	Qubiotech	MR, SPECT	Epilepsy	Automatic co-registration SPECT-MRI, interictal SPECT subtraction ictal image, identification of epileptogenic focus, percentage overlap of the focus	During: perception aid (prompting all abnormalities/results/heatmaps), interactive decision support (shows abnormalities/results only on demand), report suggestion
Neurophet AQUA	Neurophet	MR	Mild cognitive impairment, Alzheimer's disease, dementia	Brain region segmentation, volume quantification, normative comparison, report generation, white matter hyperintensity quantification, multi-time-point analysis.	During: perception aid (prompting all abnormalities/results/heatmaps)
qER	Qure.ai	CT	Intracranial hemorrhage, infarct, cranial fracture, pneumocephalus, hydrocephalus, midline shift, cerebral atrophy	Brain pathology detection, mobile notifications, worklist prioritisation, report generation, traumatic brain injury tracking	Before: adapting worklist order, flagging acute findings During: perception aid (prompting all abnormalities/results/heatmaps), interactive decision support (shows abnormalities/results only on demand), report suggestion
QyScore	Qynapse	MR, PET	Dementia, Multiple sclerosis, Alzheimer's disease, neuro-degenerative diseases	Brain region quantification, comparison to reference population, white matter hyperintensities segmentation and evolution in time and space, report generation	During: perception aid (prompting all abnormalities/results/heatmaps), report suggestion After: diagnosis verification
Rapid MRI	RapidAI	MR	Stroke		
SyMRI NEURO	SyntheticMR	MR	Multiple sclerosis, atrophy, dementia, neurogenerative diseases	Contrast image synthesis, brain region segmentation, degree of myelination,	
TRACE4AD	DeepTrace Technologies Srl	MR	Alzheimer’s disease dementia	Mild cognitive impairment (MCI) subject risk score (low risk vs high risk) of being affected by or progressing to Alzheimer's disease dementia within 24 months, cognitive, functional and behavioral deficits of the subject, subject gray matter volume and images with normative comparison, best voxel-based map predictors overlayed on the subject gray matter, best neuropsychological predictors	During: interactive decision support (shows abnormalities/results only on demand), report suggestion After: diagnosis verification
Viz ANEURYSM	Viz.ai	CT	Cerebral aneurysm	Aneurysm detection, notification	
Viz ICH	Viz.ai	CT	ICH		
SwiftMR	AIRS Medical	MR	Not applicable	MRI scan acceleration, MRI image enhancement	
Jazz	AI Medical	MR	Multiple sclerosis (MS), brain metastasis	Brain lesion detection, volume quantification	During: interactive decision support (shows abnormalities/results only on demand), report suggestion
VUNO Med-DeepBrain‚	VUNO	MR	Atrophy, Dementia	Brain parcellation, Atrophy quantification	During: perception aid (prompting all abnormalities/results/heatmaps), interactive decision support (shows abnormalities/results only on demand), report suggestion

*AI* Artificial intelligence, *ASPECTS* Alberta Stroke Program Early CT Score, *CSS* central spinal stenosis, *CT* computed tomography, *HDVS* hyperdense vessel sign, *ICA* internal carotid artery, *ICH* intracranial haemorrhage, *M1* first segment of the middle cerebral artery, *M2* second segment of the middle cerebral artery, *MCI* mild cognitive impairment, *MR* magnetic resonance, *MRI* magnetic resonance imaging, MS multiple sclerosis, *PET* positron emission tomography, *SISCOM* subtraction ictal single-photon emission computed tomography co-registered to magnetic resonance imaging, *SPECT* single-photon emission computed tomography, *TBI* traumatic brain injury, *WMH* white matter hyperintensities

Acquisition-embedded AI is already shipped on scanners. Siemens Deep Resolve (including Boost/Sharp), General Electric AIR Recon DL, and Philips SmartSpeed are marketed to accelerate MRI and improve signal-to-noise ratio, with paediatric protocol packs and claims of faster examinations. Independent paediatric evidence is emerging but remains limited to small, mostly single-centre studies focused on image quality rather than patient outcomes such as sedation rates. Early paediatric neuroimaging evaluations report feasibility of deep-learning reconstruction for synthetic MRI and accelerated 3-D T1-weighted protocols, with improved image quality and reduced acquisition times; however, designs are largely observational and underpowered for clinical endpoints [43, 45, 46].

Clinically, AI-driven MRI reconstruction and motion correction tools are undergoing trials to assess their potential to reduce sedation requirements. Anecdotal evidence further supports AI’s clinical value; a recent report highlighted an AI algorithm detecting subtle cortical dysplasia previously missed by human interpretation, enabling successful epilepsy surgery [24]. As such examples move increasingly into clinical practice, supported by those in the adult neuroradiology and general paediatric populations, confidence in AI will continue to grow. Over the next decade, broader participation in AI registries and clinical trials will help generate the necessary evidence for regulatory approval and wider adoption.

## Current challenges and practical hurdles

Despite promising research, significant barriers hinder clinical adoption of AI in paediatric neuroradiology. These challenges include (a) cost and access, (b) standardisation and interoperability, and (c) safety, governance, and ethics [47–49].

## Cost and access

Implementing AI in radiology requires substantial investment in software, hardware (high-performance graphical processing units (GPUs) or servers), and integration into picture archive and communications systems (PACS)/radiology information systems (RIS) workflows [50, 51]. Budget constraints in paediatric imaging departments can make acquiring advanced AI tools prohibitively expensive, particularly for smaller hospitals or low-resource settings. Available commercial AI tools often focus on high-volume adult conditions (stroke or chest imaging), limiting economic justification for smaller paediatric caseloads. Thus, disparities arise where major academic paediatric hospitals may trial AI tools, while regional centres struggle to afford them [52–55].

Internal development of bespoke paediatric AI models is equally resource intensive, demanding data scientists and computational infrastructure often unavailable in smaller centres. Demonstrating cost effectiveness is also challenging; administrators require robust evidence of efficiency improvements or clinical outcomes (e.g. reduced scanning times or misdiagnoses) to justify investment. Such evidence remains limited in paediatric neuroradiology. Moreover, AI models may require regular recalibration or retraining, incurring continuous costs, an issue that is complicated by the added variability of age-related brain development [56]. Without clear reimbursement pathways or external funding, economic barriers remain significant [47–49].

## Standardisation and interoperability

A fundamental barrier is the paucity of large, standardised datasets and established validation frameworks. While many medical imaging datasets exist [57], access can be siloed and region dependent. AI performance heavily depends on large-scale data, yet paediatric neuroimaging datasets remain fragmented and scarce [13, 44]. Contributing factors include lower patient volumes, rare conditions, stringent privacy regulations, and variability in imaging protocols. Consequently, AI models frequently train on single-centre datasets, limiting generalisability. The lack of standardised paediatric image repositories and benchmarks hinders objective algorithm comparison and development [5, 9].

Efforts like the FL-PedBrain federated learning platform (aggregating data from 19 sites for paediatric brain tumours) [44] are emerging, although they remain preliminary, and there have been explicit calls for the inclusion of paediatric data for AI research [13] and recognition of the crucial role of this inclusion [58, 59]. Interoperability also presents challenges: diverse scanner manufacturers, imaging protocols, and PACS systems complicate uniform AI deployment. AI tools developed for specific settings may underperform when transferred elsewhere unless robustly generalisable [5, 16, 50, 60, 61].

Additionally, paediatric-specific AI standards and guidelines are limited [62]. Existing validation and reporting frameworks (Food and Drug Association (FDA), Radiological Society of North America (RSNA)) do not explicitly address paediatric factors such as age stratification. Integration into existing radiology workflows further complicates matters. Radiologists expect AI results seamlessly integrated within PACS, yet achieving this requires standardised interfaces (e.g. DICOM structured reporting), which vary among vendors. Multiple AI tools might present outputs differently (visual highlights vs. textual reports), potentially fragmenting workflows. While standards such as Imaging Data Commons and Fast Healthcare Interoperability Resources are evolving, their adoption is not yet widespread. Comprehensive standardisation of datasets, evaluation metrics, and integration protocols remains essential for wider AI adoption in paediatric neuroradiology.

## Safety, governance, and ethics

Ensuring AI is safe, unbiased, and ethically implemented for children remains paramount [59, 63–65]. Algorithmic bias is a significant concern; models predominantly trained on adult data or limited paediatric populations may not generalise across diverse age groups, ethnicities, or developmental stages. This is especially of concern in mixed adult and paediatric centres where AI models (for example, intended for the triage of CT scans for intracranial bleeding in adults in the emergency department) could be accidentally used off-label in children, potentially leading to incorrect results that could falsely alarm or reassure the clinical teams [43].

Age-related anatomical variations (e.g. myelination, cranial morphology) differ significantly, increasing the risk of misclassification of normal developmental variations as pathology or vice versa [58, 59, 66]. Paediatric datasets often disproportionately represent specific demographics (tertiary referral populations, regional biases), amplifying these risks [11]. To mitigate healthcare disparities, rigorous validation across age ranges and diverse populations is essential.

Data privacy represents another challenge. Children are legally protected under stringent privacy frameworks (General Data Protection Regulation (GDPR), Health Insurance Portability and Accountability Act (HIPAA)), complicating large-scale data aggregation. Techniques like federated learning (where models train without raw data sharing) show promise but remain under refinement [44, 51, 55].

The complexity of these issues is compounded by the need for regulatory approval. Agencies (FDA, conformité européenne (CE) marking) have been cautious regarding paediatric-specific AI device authorisations; only approximately 3–4% of FDA-cleared radiology AI devices currently hold explicit paediatric indications [11, 62]. Consequently, many tools applied clinically in paediatrics remain “off-label”, necessitating rigorous internal validation. Professional bodies like the American College of Radiology advocate explicit labelling of validated age groups for AI products [11, 62].

Medico-legal accountability also warrants attention. Misdiagnoses or false positive findings from AI raise liability concerns. Current guidelines emphasise AI as decision support, maintaining ultimate accountability with the interpreting radiologist. Thus, AI systems require transparency and explainability. While many methods of explainability exist [67], many advanced deep learning models are inherently opaque (“black boxes”), complicating clinical and parental acceptance. To build trust, governance frameworks involving rigorous prospective validation, continuous post-deployment monitoring, and protocols for radiologist oversight and override are necessary. Ethically, involving families and communities in AI implementation discussions (including explicit consent processes and transparent communication about AI roles) is vital [59, 66, 68–71].

Overcoming bias with diverse data, safeguarding privacy, adhering to paediatric-specific regulatory standards, and maintaining human oversight are essential to ensure safe, equitable, and ethical AI deployment in paediatric neuroradiology.

## Future potential and emerging applications

Several promising directions exist for AI to significantly impact paediatric neuroradiology. Emerging applications align with critical clinical needs, from safer imaging techniques to advanced diagnostic support. Here, we discuss future potential in key areas: (a) image optimisation and safety, (b) quantitative analysis and automated metrics, and (c) advanced clinical decision support. We also consider the (d) regulatory, reimbursement, and medico-legal pathways; (e) ethics, consent, and communication; and (f) workflow integration and clinician trust issues, which are central to adoption.

## Image optimisation and safety

AI is expected to rapidly advance image acquisition techniques, improving patient safety through reduced contrast use and radiation exposure. Synthetic contrast-enhanced MRI exemplifies this potential [72–74]. AI models trained on paired non-contrast and contrast-enhanced MRI scans can predict virtual contrast images, minimising or eliminating gadolinium-based contrast agent risks [75]. Preliminary adult studies show promising results; extending this approach to paediatrics would significantly enhance safety and comfort during follow-up scans, particularly for oncological surveillance.

AI techniques also promise substantial reductions in radiation dose for paediatric CT and PET imaging. Deep learning–based denoising and super resolution methods enable diagnostic quality images from ultra-low dose protocols, adhering closely to ALARA principles. Initial adult implementations have demonstrated dose reductions exceeding 50%; similar or greater benefits are expected in paediatrics due to higher radiosensitivity [1, 76].

Additionally, motion correction and accelerated imaging through AI could reduce sedation requirements in paediatric MRI. Prospective AI-driven motion tracking (navigator signals or real-time video tracking) and retrospective AI-based k-space corrections (e.g. DISORDER technique) [18] represent significant advances. AI-enhanced image reconstruction also markedly reduces scan duration, further reducing patient discomfort and the need for sedation. Real-time AI quality control could automatically flag and correct motion degraded sequences or dynamically adjust scanner parameters (“closed loop imaging”) [11]. These techniques together promise safer, faster, and higher quality paediatric neuroradiology exams.

## Quantitative analysis and automated volumetry

Future AI systems will increasingly deliver quantitative metrics as standard outputs, moving radiology from qualitative to precise quantitative assessment. Automated volumetric analysis could replace subjective descriptions (e.g. “mild ventriculomegaly”) with precise measurements (ventricular volume, hippocampal size, lesion growth rates). For instance, in paediatric hydrocephalus, AI could accurately quantify ventricular changes, informing shunt management decisions [16]. This is especially relevant in the paediatric context considering the need for age- and sex-stratified normative atlases, as volumetric interpretation in children requires developmental context. Similarly, AI-derived tumour volumetry can improve reproducibility in monitoring growth or therapeutic response, while aiding neurosurgical planning through enhanced lesion delineation [1, 77, 78].

AI could also generate developmental metrics such as brain maturation scores by comparing individual imaging to normative paediatric atlases [28]. Such quantitative assessments may help in the early identification of developmental delays or disorders like autism, prompting timely intervention.

Radiomics-based predictive analytics represent another emerging application [8, 17, 78]. AI-generated radiomic signatures could predict tumour histology or genetic mutations non-invasively, informing clinical decisions and reducing invasive diagnostic procedures [17, 79–81]. For instance, predictive AI models might estimate the likelihood of specific tumour subtypes (e.g. pilocytic astrocytoma vs. medulloblastoma), providing probabilistic guidance before surgical intervention.

Longitudinal analysis powered by AI will become commonplace, tracking changes in lesion burden, myelination, or subtle disease progression (e.g. neurofibromatosis, tuberous sclerosis), ensuring consistent disease surveillance and personalised patient management [11, 78].

## Advanced clinical decision support

AI’s ultimate aim in paediatric neuroradiology is sophisticated decision support, enhancing diagnostic accuracy and clinical management. Radiomics and radiogenomics are poised to become routine clinical tools. AI-driven models predicting tumour molecular markers (e.g. H3K27M mutation in diffuse midline gliomas) or prognostic factors could provide early guidance on therapy selection or trial enrolment, even before biopsy results [17, 19, 80, 82]. Machine learning could also stratify risk or predict outcomes, such as post-surgical seizure freedom in epilepsy [15, 24] or neurodevelopmental prognosis post hypoxic ischaemic injury [27], facilitating early targeted interventions.

Automated detection systems will mature, providing real-time second reader capabilities. AI trained on abusive head trauma imaging patterns, integrating CT and retinal findings, could improve timely recognition and diagnosis. Early prototypes already indicate feasibility in flagging high suspicion abuse cases for further review [26, 83]. Similarly, AI may predict therapeutic responses in epilepsy through combined imaging and EEG analysis [84, 85].

Workflow prioritisation through AI-driven triage is another promising area. Urgent findings (e.g. unexpected cerebral herniation or stroke) detected by AI could trigger immediate alerts, expediting clinical response. Natural language processing could further streamline reporting, drafting preliminary radiology reports for expert verification, thus enhancing efficiency [86, 87].

## Regulatory, reimbursement, and medico-legal aspects

Future systems must be designed as high-risk medical devices with clear intended use, age range, and paediatric performance claims, rather than extrapolated adult data, in line with the new multi-society implementation framework [56]. Transparent description of datasets, subgroup performance, and paediatric safety ratings will be a precondition for approval, reimbursement, and avoidance of off-label use. Studies should move beyond accuracy metrics to include prospective implementation and health-economic outcomes, and AI outputs and user interactions should be logged in an auditable way to support quality assurance, incident review, and medico-legal scrutiny [56].

## Ethics, consent, and communication

The framework emphasises child-centred deployment, public and patient involvement, and AI literacy for all staff in paediatric imaging [56]. In paediatric neuroradiology, consent processes should explicitly state when and how AI is used, with information adapted to the developmental stage. Future work should include participatory studies with children, young people, and families on how to present data in ways that support shared decision-making without increasing anxiety [59, 65].

## Workflow integration and clinician trust

Adoption will depend on tools that integrate into PACS/RIS with defined workflow touchpoints (triage, protocol selection, quantitative add-ons, MDT visualisation), coupled with phased pilots and post-market surveillance [56]. Systems should expose uncertainty, provide simple interfaces, and allow radiologists to accept, modify, or reject outputs, with feedback loops and monitoring dashboards for ongoing performance and bias auditing. Targeted AI education for paediatric neuroradiologists, radiographers, and referrers will be essential to build appropriate trust and avoid over-reliance.

Crucially, these advances maintain radiologists’ central oversight role. A “human-centric AI” approach ensures radiologists validate AI outputs contextually, thereby augmenting rather than replacing expert clinical judgement [11, 59, 65]. AI thus expands paediatric neuroradiologists’ capabilities, enabling more comprehensive multi-disciplinary patient care and deeper diagnostic insight.

## Discussion and limitations

There is great hope that AI in paediatric and fetal neuroradiology will transition from an emerging research topic towards clinical maturity and we have discussed several avenues and potential benefits in this review. However, significant efforts are still required to realise its full potential. Despite much promise, adoption is constrained by absent prospective trials and lack of clarity regarding paediatric indications for CE-marked or FDA-cleared tools, as shown in prior device surveys [43, 88]. Off-label deployment remains a risk, with inconsistent labelling of target populations and suitability for children [43].

This review demonstrates that while numerous studies have established technical feasibility for AI applications, from tumour segmentation to lesion identification and outcome prediction, routine clinical adoption remains limited (Tables 1 and 2). An important consideration is that the old adage that children are not simply “small adults” is especially true in clinical AI development. Algorithms must be explicitly trained and validated on paediatric datasets; adult-trained AI models frequently underperform in paediatric populations due to distinct developmental and disease presentation differences; however, we acknowledge that work is still needed to establish the minimum dataset size/diversity needed for generalisability [11, 56]. Encouragingly, paediatric-focused collaborations (e.g. data-sharing consortia, specialised conferences, American College of Radiology and the European Society of Paediatric Radiology Paediatric AI Workgroups) increasingly acknowledge this distinction, advocating for tailored solutions. Expanding curated and annotated paediatric datasets and leveraging federated learning or other privacy-preserving technologies are essential next steps to facilitate AI training without compromising privacy [13, 44]. Over the next decade, integration into registries, prospective multi-centre evaluations, and harmonised labelling will be essential to move beyond anecdotal reports. This will include systematic assessment of vendor-provided reconstruction tools in reducing sedation requirements, federated and privacy-preserving training to overcome data scarcity, and benchmarking of paediatric-specific diagnostic AI against clinical outcomes. Without these steps, the gap between technical capability and safe, regulated clinical adoption will persist.

Addressing identified barriers (cost, standardisation, ethics) requires multi-faceted solutions. Sustainable economic models involving research grants, public-private partnerships, or demonstrated cost effectiveness must be developed. Standardisation initiatives should continue, incorporating paediatric-specific guidelines into validation and reporting frameworks, including explicit age-based labelling of AI products [62]. Regulatory bodies could introduce incentives analogous to paediatric pharmaceutical legislation, encouraging AI developers to validate and market paediatric indications explicitly.

Foe clinical adoption to be successful, usability and trust remain critical. Active involvement of paediatric neuroradiologists in AI design e promotes clinical relevance, smooth integration into workflows, and improved acceptance. User-friendly interfaces, intuitive visualisations (such as AI-annotated MRI overlays), and explainable AI outputs facilitate clinician adoption. Ongoing education and training in interpreting AI results and understanding limitations are equally important. Within multi-disciplinary paediatric teams, neuroradiologists increasingly serve as critical interpreters of AI-derived insights for clinical colleagues, emphasising the importance of maintaining rigorous standards and healthy scepticism. Discrepancies between AI and human assessments should be actively analysed as learning opportunities, either highlighting subtle findings overlooked by radiologists or exposing AI errors.

Our review has several limitations. The review was narrative, rather than systematic, which may introduce selection bias and limit reproducibility. Screening was conducted by a single reviewer, which may risk omissions or subjective inclusion bias. Inclusion of preprints not only increases coverage but also introduces the risk of citing non–peer-reviewed, unverified findings. Although our review maps a decade of research, the literature base in paediatric neuroradiology is still sparse and heterogeneous, making strong generalisations difficult. Included studies often have small sample sizes (tens to hundreds of cases), and reliance on proof-of-concept and single-centre studies limits the strength of clinical translation. Furthermore, it is possible that many failed experiments or attempts at implementation have not been published, adding the potential for publication bias to the issues above. There also remains a gap between research applications and clinical translation, with limited tools commercially available specifically for children. In this context, the lack of prospective, multi-centre validation remains a recurring issue for the field.

## Conclusion

In this review, we have considered 10 years of paediatric and fetal neuroradiology AI literature. However, looking forward a decade, success could lead to AI systems routinely detecting subtle brain malformations, optimising imaging protocols to reduce radiation exposure, or predicting tumour biology preoperatively. Such advances would profoundly improve clinical care, facilitating precise, proactive, and personalised management of paediatric neurological disorders. Thoughtful integration of AI, with continued primacy of expert human judgement, promises substantial benefits, ensuring improved outcomes and experiences for children and families. While challenges exist, the potential reward of healthier children enabled by smarter imaging certainly justifies dedicated pursuit of this goal.

To this end, paediatric neuroradiology should align with the new multi-society implementation framework for paediatric radiology [56], which organises action around four pillars: regulation and purchasing, implementation and integration, interpretation and post-market surveillance, and education. Practically, we call for (i) multi-centre data-sharing initiatives with paediatric-specific safety ratings and transparent reporting of dataset composition and subgroup performance; (ii) prospective, workflow-embedded studies that include health-economic and patient-centred outcomes; and (iii) structured AI literacy and governance programmes, with the involvement of children, young people, and families, to support trustworthy use.

## Data Availability

No datasets were generated or analysed during the current study.

## References

[1] Pringle C, Kilday J-P, Kamaly-Asl I, Stivaros SM (2022) The role of artificial intelligence in paediatric neuroradiology. Pediatr Radiol 52:2159–2172 35347371 10.1007/s00247-022-05322-w PMC9537195

[2] Bailey CR, Bailey AM, McKenney AS, Weiss CR (2022) Understanding and appreciating burnout in radiologists. Radiographics 42:E137–E139 35839137 10.1148/rg.220037

[3] Bastian MB, Fröhlich L, Wessendorf J et al (2024) Prevalence of burnout among German radiologists: a call to action. Eur Radiol 34:5588–5594 38345608 10.1007/s00330-024-10627-5 PMC11364704

[4] Fawzy NA, Tahir MJ, Saeed A et al (2023) Incidence and factors associated with burnout in radiologists: a systematic review. Eur J Radiol Open 11:100530 37920681 10.1016/j.ejro.2023.100530 PMC10618688

[5] Kelly BS, Clifford SM, Judge CS, et al (2024) A survey of paediatric radiology artificial intelligence. medRxiv 2024.09.19.24313885. 10.1101/2024.09.19.24313885

[6] Kelly BS, Judge C, Bollard SM et al (2022) Radiology artificial intelligence: a systematic review and evaluation of methods (RAISE). Eur Radiol. 10.1007/s00330-022-08784-6 35420305 PMC9668941

[7] Ryan M-L, O’Donovan T, McNulty JP (2021) Artificial intelligence: the opinions of radiographers and radiation therapists in Ireland. Radiography 27:S74–S82 34454835 10.1016/j.radi.2021.07.022

[8] da Rocha JLD, Lai J, Pandey P et al (2025) Artificial intelligence for neuroimaging in pediatric cancer. Cancers (Basel) 17:622 40507257 10.3390/cancers17111776 PMC12153587

[9] Martin D, Tong E, Kelly B et al (2021) Current perspectives of artificial intelligence in pediatric neuroradiology: an overview. Front Radiol 1:713681 37492174 10.3389/fradi.2021.713681 PMC10365125

[10] Otjen JP, Moore MM, Romberg EK et al (2022) The current and future roles of artificial intelligence in pediatric radiology. Pediatr Radiol 52:2065–2073 34046708 10.1007/s00247-021-05086-9

[11] Bhatia A, Khalvati F, Ertl-Wagner BB (2024) Artificial intelligence in the future landscape of pediatric neuroradiology: opportunities and challenges. AJNR Am J Neuroradiol 45:549–553 38176730 10.3174/ajnr.A8086 PMC11288527

[12] Yu X, Li S, Mai W et al (2024) Pediatric diffuse intrinsic pontine glioma radiotherapy response prediction: MRI morphology and T2 intensity-based quantitative analyses. Eur Radiol 34:7962–7972 38907098 10.1007/s00330-024-10855-9 PMC11557687

[13] Muralidharan V, Burgart A, Daneshjou R, Rose S (2023) Recommendations for the use of pediatric data in artificial intelligence and machine learning ACCEPT-AI. NPJ Digit Med 6:166 37673925 10.1038/s41746-023-00898-5 PMC10482936

[14] Baethge C, Goldbeck-Wood S, Mertens S (2019) SANRA—a scale for the quality assessment of narrative review articles. Res Integr Peer Rev 4:5 30962953 10.1186/s41073-019-0064-8 PMC6434870

[15] Jeong J-W, Lee M-H, Kuroda N et al (2022) Multi-scale deep learning of clinically acquired multi-modal MRI improves the localization of seizure onset zone in children with drug-resistant epilepsy. IEEE J Biomed Health Inform 26:5529–5539 35925854 10.1109/JBHI.2022.3196330 PMC9710730

[16] Quon JL, Han M, Kim LH et al (2021) Artificial intelligence for automatic cerebral ventricle segmentation and volume calculation: a clinical tool for the evaluation of pediatric hydrocephalus. J Neurosurg Pediatr 27:131–138 33260138 10.3171/2020.6.PEDS20251 PMC9707365

[17] Zhang M, Wong SW, Wright JN et al (2022) MRI radiogenomics of pediatric medulloblastoma: a multicenter study. Radiology 304:406–416 35438562 10.1148/radiol.212137 PMC9340239

[18] Vecchiato K, Egloff A, Carney O, et al (2020) Evaluation of DISORDER: retrospective image motion correction for volumetric brain MRI in a pediatric setting. medRxiv 2020.09.09.20190777. 10.1101/2020.09.09.20190777 PMC8040996 33602745

[19] Chang F-C, Wong T-T, Wu K-S et al (2021) Magnetic resonance radiomics features and prognosticators in different molecular subtypes of pediatric medulloblastoma. PLoS One 16:e0255500 34324588 10.1371/journal.pone.0255500 PMC8321137

[20] Yepes-Calderon F, McComb JG (2022) Accurate image-based CSF volume calculation of the lateral ventricles. Sci Rep 12:12115 35840587 10.1038/s41598-022-15995-w PMC9287564

[21] Hatamizadeh A, Nath V, Tang Y, et al (2022) Brainlesion: glioma, multiple sclerosis, stroke and traumatic brain injuries, 7th International Workshop, BrainLes 2021, Held in Conjunction with MICCAI 2021, Virtual Event, September 27, 2021, Revised Selected Papers, Part I. Lect Notes Comput Sci 272–284. 10.1007/978-3-031-08999-2_22

[22] Spitzer H, Ripart M, Whitaker K et al (2022) Interpretable surface-based detection of focal cortical dysplasias: a multi-centre epilepsy lesion detection study. Brain 145:3859–3871 35953082 10.1093/brain/awac224 PMC9679165

[23] Wagstyl K, Whitaker K, Raznahan A et al (2022) Atlas of lesion locations and postsurgical seizure freedom in focal cortical dysplasia: a MELD study. Epilepsia 63:61–74 34845719 10.1111/epi.17130 PMC8916105

[24] Ripart M, Spitzer H, Williams LZJ et al (2025) Detection of epileptogenic focal cortical dysplasia using graph neural networks. JAMA Neurol 82:397–406 39992650 10.1001/jamaneurol.2024.5406 PMC11851297

[25] Azad Z, Aiman U, Shaheen S (2024) Artificial intelligence in paediatric head trauma: enhancing diagnostic accuracy for skull fractures and brain haemorrhages. Neurosurg Rev 47:641 39294484 10.1007/s10143-024-02897-w

[26] Heo S, Ha J, Jung W et al (2022) Decision effect of a deep-learning model to assist a head computed tomography order for pediatric traumatic brain injury. Sci Rep 12:12454 35864281 10.1038/s41598-022-16313-0 PMC9304372

[27] Gui L, Loukas S, Lazeyras F et al (2019) Longitudinal study of neonatal brain tissue volumes in preterm infants and their ability to predict neurodevelopmental outcome. Neuroimage 185:728–741 29908311 10.1016/j.neuroimage.2018.06.034

[28] Smyser CD, Dosenbach NUF, Smyser TA et al (2016) Prediction of brain maturity in infants using machine-learning algorithms. Neuroimage 136:1–9 27179605 10.1016/j.neuroimage.2016.05.029 PMC4914443

[29] Khalighi S, Reddy K, Midya A et al (2024) Artificial intelligence in neuro-oncology: advances and challenges in brain tumor diagnosis, prognosis, and precision treatment. NPJ Precis Oncol 8:80 38553633 10.1038/s41698-024-00575-0 PMC10980741

[30] Wu J, Chen L, Li Z, et al (2025) 3D isotropic high-resolution fetal brain MRI reconstruction from motion corrupted thick data based on physical-informed unsupervised learning. IEEE J Biomed Heal Inform PP:1–14. 10.1109/jbhi.2025.3586049 40663667

[31] Lajous H, Roy CW, Hilbert T et al (2022) A fetal brain magnetic resonance acquisition numerical phantom (FaBiAN). Sci Rep 12:8682 10.1038/s41598-022-10335-4 PMC9127105 35606398

[32] Ciceri T, Squarcina L, Giubergia A et al (2023) Review on deep learning fetal brain segmentation from magnetic resonance images. Artif Intell Med 143:102608 10.1016/j.artmed.2023.102608 37673558

[33] Khalili N, Turk E, Benders MJNL et al (2019) Automatic extraction of the intracranial volume in fetal and neonatal MR scans using convolutional neural networks. NeuroImage: Clinical 24:102061 31835284 10.1016/j.nicl.2019.102061 PMC6909142

[34] Zhao L, Asis-Cruz JD, Feng X et al (2022) Automated 3D fetal brain segmentation using an optimized deep learning approach. AJNR Am J Neuroradiol 43:448–454 35177547 10.3174/ajnr.A7419 PMC8910820

[35] Lim A, Lo J, Wagner MW et al (2023) Motion artifact correction in fetal MRI based on a generative adversarial network method. Biomed Signal Process Control 81:104484

[36] Yaqub M, Kelly B, Papageorghiou AT, Noble JA (2017) A deep learning solution for automatic fetal neurosonographic diagnostic plane verification using clinical standard constraints. Ultrasound Med Biol 43:2925–2933 28958729 10.1016/j.ultrasmedbio.2017.07.013

[37] Xie H, Wang N, He M et al (2020) Using deep learning algorithms to classify fetal brain ultrasound images as normal or abnormal. Ultrasound Obstet Gynecol. 10.1002/uog.21967 31909548

[38] YYun HJ, Lee H-J, You S et al (2025) Deep learning–based brain age prediction using MRI to identify fetuses with cerebral ventriculomegaly. Radiol Artif Intell 7:e240115 39969279 10.1148/ryai.240115 PMC11950871

[39] Shen L, Zheng J, Lee EH et al (2022) Attention-guided deep learning for gestational age prediction using fetal brain MRI. Sci Rep 12:1408 35082346 10.1038/s41598-022-05468-5 PMC8791965

[40] Kwon H, You S, Yun HJ et al (2024) The role of cortical structural variance in deep learning-based prediction of fetal brain age. Front Neurosci 18:1411334 38846713 10.3389/fnins.2024.1411334 PMC11153753

[41] Wilson S, Yun HJ, Sadhwani A et al (2025) Foetal cortical expansion is associated with neurodevelopmental outcome at 2-years in congenital heart disease: a longitudinal follow-up study. EBioMedicine 114:105679 40158387 10.1016/j.ebiom.2025.105679 PMC11994330

[42] Ouyang M, Whitehead MT, Mohapatra S et al (2024) Machine-learning based prediction of future outcome using multimodal MRI during early childhood. Semin Fetal Neonatal Med 29:101561 10.1016/j.siny.2024.101561 PMC11654837 39528363

[43] Kelly BS, Lee J, Antram E, et al (2025) Safe for kids? AI medical devices in radiology overlook paediatric suitability. Eur Radiol 1–8. 10.1007/s00330-025-11975-6 41060417

[44] Lee EH, Han M, Wright J et al (2024) An international study presenting a federated learning AI platform for pediatric brain tumors. Nat Commun 15:7615 39223133 10.1038/s41467-024-51172-5 PMC11368946

[45] Antonissen N, Tryfonos O, Houben IB et al (2025) Artificial intelligence in radiology: 173 commercially available products and their scientific evidence. Eur Radiol 11. 10.1007/s00330-025-11830-8 PMC12711992 40707732

[46] An FDA guide on indications for use and device reporting of artificial intelligence-enabled devices: significance for pediatric use. https://brandon-j-nelson.com/posts/assets/Nelson%20et%20al.%20-%202023%20-%20An%20FDA%20Guide%20on%20Indications%20for%20Use%20and%20Device%20Rep.pdf. Accessed 22 Jul 2024 10.1016/j.jacr.2023.06.004 37400046

[47] Brady AP, Allen B, Chong J et al (2024) Developing, purchasing, implementing and monitoring AI tools in radiology: practical considerations. A multi-society statement from the ACR, CAR, ESR, RANZCR & RSNA. Can Assoc Radiol J 75:226–244 38251882 10.1177/08465371231222229

[48] Brady AP, Loewe C, Brkljacic B et al (2025) Guidelines and recommendations for radiologist staffing, education and training. Insights Imaging 16:57 40074928 10.1186/s13244-025-01926-6 PMC11904080

[49] Brady AP, Bello JA, Derchi LE et al (2021) Radiology in the era of value-based healthcare: a multi-society expert statement from the ACR, CAR, ESR, IS3R, RANZCR, and RSNA. Can Assoc Radiol J 72:208–214 33345576 10.1177/0846537120982567

[50] Harvey H, Glocker B (2019) Artificial intelligence in medical imaging, opportunities, applications and risks. 61–72. 10.1007/978-3-319-94878-2_6

[51] Willemink MJ, Koszek WA, Hardell C et al (2020) Preparing medical imaging data for machine learning. Radiology 295:4–15 32068507 10.1148/radiol.2020192224 PMC7104701

[52] He J, Baxter SL, Xu J et al (2019) The practical implementation of artificial intelligence technologies in medicine. Nat Med 25:30–36 30617336 10.1038/s41591-018-0307-0 PMC6995276

[53] Recht MP, Dewey M, Dreyer K et al (2020) Integrating artificial intelligence into the clinical practice of radiology: challenges and recommendations. Eur Radiol 30:3576–3584 32064565 10.1007/s00330-020-06672-5

[54] Daye D, Wiggins WF, Lungren MP et al (2022) Implementation of clinical artificial intelligence in radiology: who decides and how? Radiology 305:555–563 35916673 10.1148/radiol.212151 PMC9713445

[55] Kelly BS, Quinn C, Belton N et al (2023) Cybersecurity considerations for radiology departments involved with artificial intelligence. Eur Radiol 33:8833–8841 37418025 10.1007/s00330-023-09860-1 PMC10667413

[56] Shelmerdine SC, Naidoo J, Kelly BS, et al (2025) AI implementation in pediatric radiology for patient safety: a multi-society statement from the ACR, ESPR, SPR, SLARP, AOSPR, SPIN. Pediatr Radiol 1–14. 10.1007/s00247-025-06386-0 41288670

[57] Jiménez-Sánchez A, Avlona N-R, Boer S de, et al (2025) In the picture: medical imaging datasets, artifacts, and their living review. Proc 2025 ACM Conf Fairness, Account, Transpar 511–531. 10.1145/3715275.3732035

[58] Shelmerdine SC (2024) Revolutionising paediatric radiology: the future impact of artificial intelligence. Eur Radiol 34:2294–2296 37819275 10.1007/s00330-023-10288-w

[59] Lee L, Salami RK, Martin H et al (2024) How I would like AI used for my imaging”: children and young persons’ perspectives. Eur Radiol. 10.1007/s00330-024-10839-9 38900281 PMC11557655

[60] Katal S, York B, Gholamrezanezhad A (2024) AI in radiology: from promise to practice − a guide to effective integration. Eur J Radiol 181:111798 39471551 10.1016/j.ejrad.2024.111798

[61] dos Santos DP, Dietzel M, Baessler B (2021) A decade of radiomics research: are images really data or just patterns in the noise? Eur Radiol 31:1–4 32767103 10.1007/s00330-020-07108-w PMC7755615

[62] Brewster RCL, Nagy M, Wunnava S, Bourgeois FT (2025) US FDA approval of pediatric artificial intelligence and machine learning–enabled medical devices. JAMA Pediatr 179:212–214 39680415 10.1001/jamapediatrics.2024.5437 PMC11791695

[63] Shelmerdine SC (2024) Rethinking our relationship with AI: for better or worse, richer or poorer? Eur Radiol. 10.1007/s00330-024-11007-9 39095603

[64] O’Neil C, Gunn H (2020) Ethics of artificial intelligence. 237–270. 10.1093/oso/9780190905033.003.0009

[65] Kelly B, Kirwan A, Quinn M et al (2023) The ethical matrix as a method for involving people living with disease and the wider public (PPI) in near-term artificial intelligence research. Radiography. 10.1016/j.radi.2023.03.009 37062673

[66] Ciet P, Eade C, Ho M-L et al (2024) The unintended consequences of artificial intelligence in paediatric radiology. Pediatr Radiol 54:585–593 37665368 10.1007/s00247-023-05746-y

[67] Borys K, Schmitt YA, Nauta M et al (2023) Explainable AI in medical imaging: an overview for clinical practitioners – beyond saliency-based XAI approaches. Eur J Radiol 162:110786 36990051 10.1016/j.ejrad.2023.110786

[68] Stogiannos N, Malik R, Kumar A et al (2023) Black box no more: a scoping review of AI governance frameworks to guide procurement and adoption of AI in medical imaging and radiotherapy in the UK. Br J Radiol 96:20221157 37747285 10.1259/bjr.20221157 PMC10646619

[69] Gastounioti A, Kontos D (2020) Is it time to get rid of black boxes and cultivate trust in AI? Radiol Artif Intell 2:e200088 32510520 10.1148/ryai.2020200088 PMC7259191

[70] Ursin F, Timmermann C, Steger F (2021) Explicability of artificial intelligence in radiology: is a fifth bioethical principle conceptually necessary? Bioethics. 10.1111/bioe.12918 34251687

[71] Malamateniou C, Knapp KM, Pergola M et al (2021) Artificial intelligence in radiography: where are we now and what does the future hold? Radiography 27:S58–S62 34380589 10.1016/j.radi.2021.07.015

[72] Kim E, Cho H-H, Cho SH et al (2022) Accelerated synthetic MRI with deep learning–based reconstruction for pediatric neuroimaging. AJNR Am J Neuroradiol 43:1653–1659 10.3174/ajnr.A7664 PMC9731246 36175085

[73] Nagaraj UD, Meineke J, Sriwastwa A et al (2025) Evaluation of synthetic images derived from a neural network in pediatric brain magnetic resonance imaging. Magn Reson Imaging 121:110427 40389019 10.1016/j.mri.2025.110427

[74] Pasquini L, Napolitano A, Pignatelli M et al (2022) Synthetic post-contrast imaging through artificial intelligence: clinical applications of virtual and augmented contrast media. Pharmaceutics 14:2378 36365197 10.3390/pharmaceutics14112378 PMC9695136

[75] Gräfe D, Simion S-H, Rosolowski M et al (2023) Brain deposition of gadobutrol in children—a cross-sectional and longitudinal MRI T1 mapping study. Eur Radiol 33:4580–4588 36520178 10.1007/s00330-022-09297-y PMC10289941

[76] Ng CKC (2022) Artificial intelligence for radiation dose optimization in pediatric radiology: a systematic review. Children 9:1044 35884028 10.3390/children9071044 PMC9320231

[77] Huang J, Shlobin NA, Lam SK, DeCuypere M (2022) Artificial intelligence applications in pediatric brain tumor imaging: a systematic review. World Neurosurg 157:99–105 34648981 10.1016/j.wneu.2021.10.068

[78] Pacchiano F, Tortora M, Doneda C et al (2024) Radiomics and artificial intelligence applications in pediatric brain tumors. World J Pediatr 20:747–763 10.1007/s12519-024-00823-0 PMC11402857 38935233

[79] Ende E, Ryan S, Crain MA, Makary MS (2023) Artificial intelligence, augmented reality, and virtual reality advances and applications in interventional radiology. Diagnostics 13:892 36900036 10.3390/diagnostics13050892 PMC10000832

[80] (ESR) ES of R (2015) Medical imaging in personalised medicine: a white paper of the research committee of the European Society of Radiology (ESR). Insights Imaging 6:141–155 10.1007/s13244-015-0394-0 PMC4376812 25763994

[81] Saxena S, Jena B, Gupta N et al (2022) Role of artificial intelligence in radiogenomics for cancers in the era of precision medicine. Cancers 14:2860 35740526 10.3390/cancers14122860 PMC9220825

[82] Liu Z, Duan T, Zhang Y et al (2023) Radiogenomics: a key component of precision cancer medicine. Br J Cancer 129:741–753 37414827 10.1038/s41416-023-02317-8 PMC10449908

[83] Shahi N, Shahi AK, Phillips R et al (2021) Using deep learning and natural language processing models to detect child physical abuse. J Pediatr Surg 56:2326–2332 33838900 10.1016/j.jpedsurg.2021.03.007

[84] Tveit J, Aurlien H, Plis S et al (2023) Automated interpretation of clinical electroencephalograms using artificial intelligence. JAMA Neurol 80:805–812 37338864 10.1001/jamaneurol.2023.1645 PMC10282956

[85] Kim H, Hwang H (2021) Application of artificial intelligence (AI) and machine learning (ML) in pediatric epilepsy: a narrative review. Pediatr Med Chir 0:0–0

[86] Davendralingam N, Sebire NJ, Arthurs OJ, Shelmerdine SC (2021) Artificial intelligence in paediatric radiology: future opportunities. Br J Radiol 94:20200975 32941736 10.1259/bjr.20200975 PMC7774693

[87] Katzman BD, Pol CB, Soyer P, Patlas MN (2023) Artificial intelligence in emergency radiology: a review of applications and possibilities. Diagn Interv Imaging 104:6–10 35933269 10.1016/j.diii.2022.07.005

[88] Nelson BJ, Zeng R, Sammer MBK et al (2023) An FDA guide on indications for use and device reporting of artificial intelligence-enabled devices: significance for pediatric use. J Am Coll Radiol 20:738–741 37400046 10.1016/j.jacr.2023.06.004

